# Context-dependent extinction of threat memories: influences of healthy aging

**DOI:** 10.1038/s41598-018-31000-9

**Published:** 2018-08-22

**Authors:** Simone Battaglia, Sara Garofalo, Giuseppe di Pellegrino

**Affiliations:** 1 Centre for Studies and Research in Cognitive Neuroscience, Department of Psychology, University of Bologna, Bologna, Italy; 2 Neurology Unit, IRCCS Istituto Neurologico Mediterraneo (INM) Neuromed, Pozzilli, Italy

## Abstract

Although a substantial progress has been made in recent years on understanding the processes mediating extinction of learned threat, little is known about the context-dependent extinction of threat memories in elderly individuals. We used a 2-day differential threat conditioning and extinction procedure to determine whether young and older adults differed in the contextual recall of conditioned responses after extinction. On Day 1, conditioned stimuli were paired with an aversive electric shock in a ‘danger’ context and then extinguished in a different ‘safe’ context. On Day 2, the extinguished stimulus was presented to assess extinction recall (safe context), and threat renewal (danger context). Physiological and verbal report measures of threat conditioning were collected throughout the experiment. Skin conductance response (SCR data revealed no significant differences between age groups during acquisition and extinction of threat conditioning on Day 1. On Day 2, however, older adults showed impaired recall of extinction memory, with increased SCR to the extinguished stimulus in the ‘safe’ context, and reduced ability to process context properly. In addition, there were no age group differences in fear ratings and contingency awareness, thus revealing that aging selectively impairs extinction memories as indexed by autonomic responses. These results reveal that aging affects the capacity to use context to modulate learned responses to threat, possibly due to changes in brain structures that enable context-dependent behaviour and are preferentially vulnerable during aging.

## Introduction

Extinction of threat memories is a phenomenon that allows animals and humans to adapt their behaviour to a changing environment. During extinction, repeated presentation of the conditioned stimulus (CS) alone after Pavlovian or classical, threat conditioning (CS–unconditional stimulus (US) pairings) causes attenuation of defensive responses^1^ (see ref.^2^ for a recent review). Several key studies^3,4^ indicate that extinction does not involve permanent erasure (i.e., unlearning) of the original associative (i.e., CS-US) memory. Instead, there is converging evidence from animal^5–8^ and human^9,10^ studies that the mechanisms supporting extinction entail new learning (i.e., CS-no US) that competes, and temporarily interferes, with the expression of the original conditioning trace. During this competition, contextual information appears to be a critical regulatory factor in determining whether the original threat memory or the new extinction memory should control defensive CS responses. For example, a renewal of responding is observed^11–13^ when, after extinction in a context (Context B) different from the acquisition context (Context A), the CS is presented in the original acquisition context (Context A). This “ABA renewal effect” has been repeatedly demonstrated in both rats^14–16^ and humans^17,18^, and suggests that extinction involves just one more form of learning that is particularly context-dependent (for excellent comprehensive reviews on threat extinction and renewal, see^2,19,20^).

It is widely agreed that aging is accompanied by a cognitive decline in laboratory animals, as well as in humans^21–23^. Declines in the ability to process contextual information, and flexibly adapt behaviour to situational changes, may represent a fundamental mechanism of age-related cognitive alterations^24^. Furthermore, considerable research in animals and humans reveals that contextual regulation of extinction memory requires coordinated activity of regions of prefrontal cortex, hippocampus, and amygdala^20^. Of these, prefrontal cortex and hippocampus-dependent behaviours are preferentially vulnerable during aging, suggesting that impairments within these structures could underlie extinction deficits in advanced age^25,26^. Although a substantial progress has been made in recent years on understanding the processes mediating extinction of learned threat^2,27^, the impact of healthy aging on the context-dependent extinction of threat memories has been relatively unexplored.

Previous deficits in the extinction of escape from spatial water maze have been reported in aged rats^28,29^. However, these rats were also impaired at the initial acquisition of spatial water maze, thereby confounding clear assessment of how aging may specifically alter extinction. Recent studies have specifically demonstrated a decline of the capacity to extinguish in aged rats^30,31^, and mice^32^, associated with difficulties in contextual regulation of extinction memory in older animals. Interestingly, age-related extinction deficits occurred in the absence of impairments in the initial acquisition and expression of defensive responses to threat stimuli, thus indicating that older animals have a selective difficulty using contextual information to modulate the expression of stimulus-response contingencies^20^.

In humans, one prior study by LaBar and colleagues^33^ examined the impact of aging on the acquisition and subsequent extinction of threat conditioning using a simple conditioning paradigm conducted within a single session. LaBar *et al*.^33^, reported no age-related reduction in threat conditioning and immediate extinction, provided that awareness of the CS–US contingency and arousal, assessed by unconditioned responding, were taken into account. There is increasing evidence from the animal^34–38^ and human^10,39,40^ studies that within-session extinction (i.e., extinction conducted immediately after threat conditioning or short-term extinction) and between-session extinction recall (e.g., long-term extinction memory) involve different mechanisms and neurobiological substrates. To date, however, no prior study has directly examined age-related differences in delayed recall of extinction memory, and the contextual dependency of long-term extinction recall in young and older adults.

**Figure 1:**
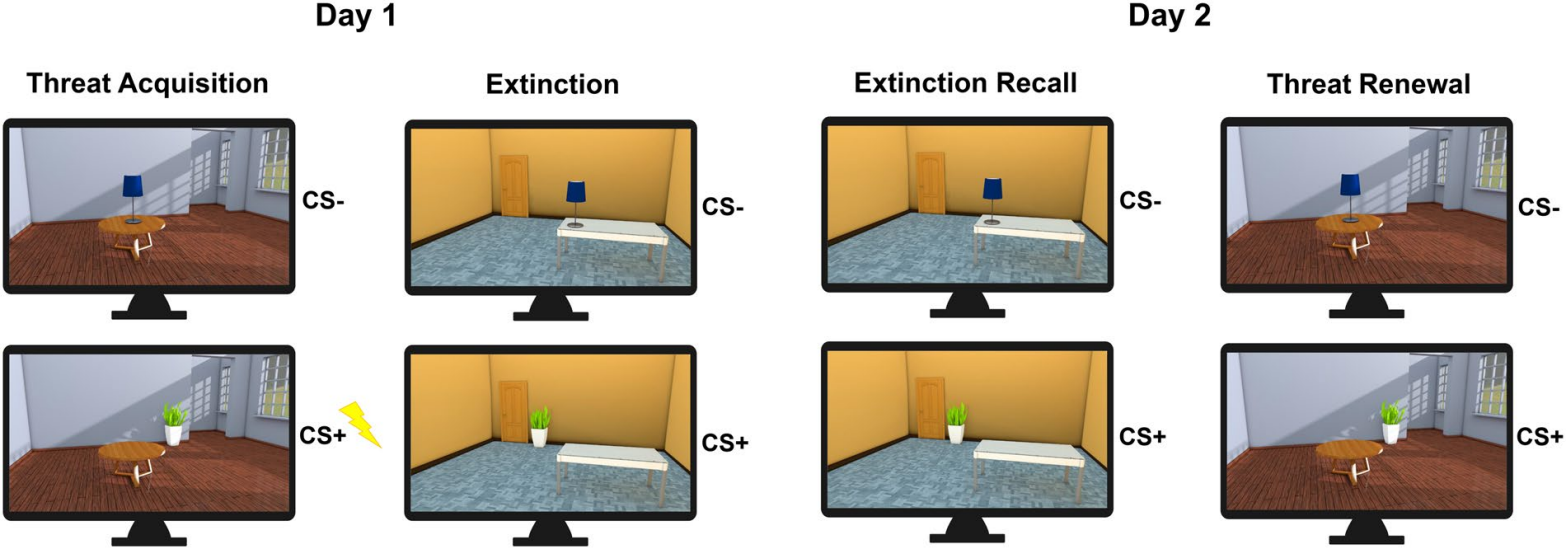
Stimuli and experimental design. Threat acquisition and extinction were established on the first day (Day 1). Participants were threat conditioned in the danger context, in which the conditioned stimulus (CS+) was associated with a shock pulse on 60% of trials, while the CS− was not associated with any consequence. Extinction followed this phase, during which both CSs were presented within the safe context and none of them was associated with the shock pulse. Extinction recall and threat renewal were administered on the second day (Day 2). The recall of extinction was tested presenting the conditioned stimuli (CSs) within the safe context (in which extinction occurred on the first day). Subsequently, renewal of threat was tested presenting CSs within the danger context (in which the threat association was learned on the first day). On the second day, all CSs were presented in absence of the shock pulse.

To test for context-dependent recall of extinction memory in aging, we used a 2-day differential threat conditioning and extinction procedure, modified from that previously described by Milad and colleagues^39,41^ (see Fig. 1). This protocol incorporates a temporal delay (24 hr) between extinction training and subsequent probing of extinction and threat memories, thus providing a more ecological test of long-term extinction memories in young and older adults. On Day 1, subjects received conditioning followed by extinction, with pictures of common objects as CSs, and electric shock as the US. To manipulate context, we presented visual CSs embedded within pictures of two distinct rooms, such that, on Day 1, threat acquisition and extinction training were performed in contexts A and B, respectively. On Day 2, participants were presented with two additional phases: extinction recall, and threat renewal, in context B (extinction context) and context A (conditioning context), respectively. No US was delivered on Day 2. Physiological (skin conductance) and verbal report measures of threat conditioning were collected throughout the experiment.

Consistent with prior aging studies in humans^33^ and non-human mammals^30–32^, we hypothesized no significant age group differences during acquisition and extinction of threat conditioning on Day 1. However, we expected that, compared to young adults, older adults would show a selective deficit in contextual processing of extinction memory on Day 2. The results of the present study should thus yield insights into age-associated changes in the extinction of threat memories and the mechanisms that enable context-dependent behaviour.

## Methods

### Participants

A total of 48 right-handed healthy adults participated in the study. Participants were divided into two age groups: twenty-four young adults (12 female; mean age = 24.79 years, SD = 3.59 years; age range: 20–30 years; mean education = 14.45 years, SD = 2.32 years), and twenty-four older adults (12 female; mean age = 66.12 years, SD = 7.60; age range: 60–70 years; mean education = 13.33 years, SD = 2.41 years). The young group was composed of Bologna University students recruited through campus advertisements, whereas the old group was recruited through a referral from the Center for Studies and Research in Cognitive Neuroscience of Bologna University, where the study was conducted, or other referral sources. Prior to participation, subjects were screened to ensure that they had no history of neurological, psychiatric, or cardiovascular conditions. None of the participants were taking any medication affecting the central nervous system regularly. All participants had normal or corrected-to-normal vision. The two groups were matched for level of education (t(1,23) = 1.453; p = 0.159). It is widely known that anxiety and depression may affect SCR in classical conditioning^42^. To account for such variability, levels of anxiety and depression were measured by means of the State-Trait Anxiety Inventory^43^, and the Hospital Anxiety and Depression Scale^44^. The two groups did not show any significant difference in terms of anxiety (young group mean = 39.14, SD = 5.32 years; old group mean = 36.83 years, SD = 5.83 years; t(1,23) = 1.386; p = 0.179), and depression (young group mean = 4.68, SD = 1.89 years; old group mean = 5.37 years, SD = 2.97 years; t(1,23) = −0.939; p = 0.357).

The study was conducted in accordance with the ethical principles of the World Medical Association Declaration of Helsinki and was approved by the Ethics Committee of the Department of Psychology of the University of Bologna. All participants gave informed written consent to participation after being informed about the procedure of the study.

### Neuropsychological assessment

Young and older adults were given a series of standardized neuropsychological tests. The primary objective in performing these tests was to rule out the possibility that any older adult participants included in our sample were affected by age-associated cognitive deficits, rather than to assess differences between young and old groups.

The battery included tests of abstract reasoning (Raven Progressive Matrices^45^), verbal short-term and long-term memory (Verbal Span with disyllabic words, and Prose Recall^45^), selective attention (Attentional Matrices Test^45^), and executive function (Weigl’s Sorting Test^45^). Normative scores derived from a nationally representative sample of adults are available for each test. For all tests, participants’ raw scores were converted into equivalent scores^46^, adjusted for age and years of education. Equivalent score is a 5-point scale, ranging from 0 to 4, with 0 = pathological performance, 1 = borderline performance, 2–4 = normal performance. The neuropsychological testing session was held one or two days before the experimental session, and only participants who were within normal ranges were asked to participate in the experiment. Table 1 shows the means, standard deviations of the equivalent score on each test for young and older participants in the study.

**Table 1:** Neuropsychological assessment.

Test	Equivalent Scores		t(23)	p
	Young	Old		
Raven Progressive Matrices	3.91 (±0.28)	3.79 (±0.58)	0.9	0.376
Verbal Span	3.12 (±0.94)	2.66 (±0.46)	1.141	0.265
Prose Recall	3.83 (±0.38)	3.70 (±0.46)	1.269	0.216
Attentional Matrices Test	3.33 (±0.70)	3.58 (±0.65)	−1.297	0.207
Weigl’s Sorting Test	3.75 (±0.67)	3.45 (±0.77)	1.231	0.231

Means, standard deviations (in brackets) and statistical comparison (t-test) between old and young participants of the equivalent scores on each test. The battery included tests of abstract reasoning (Raven Progressive Matrices), verbal short-term and long-term memory (Verbal Span, and Prose Recall), selective visual attention (Attentional Matrices Test), and executive function (Weigl’s Sorting Test). No significant differences were found between young and older participants.

### Materials

The experiment was implemented in Matlab R2016 (The MathWorks, Inc., Natick, Massachusetts, United States) software, and ran on a Windows-based PC (Lenovo ThinkCentre Desktop Computer). Stimuli were created with Blender (Blender Foundation, Amsterdam, Netherlands) and Cinema 4D R17 software (MAXON Computer GmbH, Friedrichsdorf, Germany), and were presented on a computer screen (screen size: 43 inches; resolution: 1920 × 1080; refresh rate: 60 Hz). Context scenes consisted of images of 2 different indoor scenes (i.e., a yellow-blue room, and a grey-red room), representing the acquisition (‘danger’) context and the extinction (‘safe’) context of threat associations, respectively. For half of the participants in each age group, the acquisition context and the extinction context were the yellow-blue room and the grey-red room, respectively. Context assignment was reversed for the other half so as to counterbalance across subjects which environment was associated with a shock. Conditioned stimuli (CSs) were images of two everyday common objects, a plant and a lamp, embedded within the context scenes^39,41^. For half of the participants in each group, the reinforced CS+ and the unreinforced CS− were the plant and the lamp, respectively, and vice versa for the other half. Neutral, rather than intrinsically emotional (i.e., spiders, snakes, or angry faces), stimuli were used as CSs, because conditioned responses to very salient CSs can be confounded by the ceiling effects of the respective outcome measures.

A mildly aversive electro-tactile stimulation served as unconditioned stimulus (US). The shock pulse was generated by a Digitimer Stimulator (Model DS7, Digitimer Ltd., UK) and delivered to the participants’ left inner wrist for 200 ms. The intensity of the stimulation was determined individually by assessing the participant’s subjective evaluation in a standard work up procedure prior to threat acquisition. It was initially set at 0.5 mA and increased of 1 mA until participants reported it as a “highly annoying, but not painful” stimulation.

### SCR Recording

The skin conductance response (SCR) was recorded with two Ag/AgCl electrodes (TSD203Model; Biopac Systems, USA), filled with isotonic hyposaturated conductant and attached to the distal phalanges of the second and the third finger of participants’ left hand. A DC amplifier (Biopac EDA100C) was used while recording the SCR. A gain factor was 5 μS/V and the low-pass filter was set at 10 Hz. The analog signal was then passed through a Biopac MP-150 digital converter at a 200 Hz rate. The signal was recorded with AcqKnowledge 3.9 (BIOPAC Systems, Inc., Goleta, California) and converted to microsiemens for offline analysis.

### Procedure

The study was performed at the Center for Studies and Research in Cognitive Neuroscience of the University of Bologna, in Cesena, Italy. Participants were tested individually. They were comfortably seated in a silent and dimly lit room, and their position was centered relative to the computer screen, at 100 cm viewing distance. Electrodes for SCR recording, and for shock pulse administration were attached to the participant. The SCR was recorded continuously while participants completed the task and data were stored for offline analysis. Participants were asked to remain as quiet and still as possible during the task and to keep their attention at the center of the screen. After verifying that SCR was being properly recorded, the intensity of the shock pulse to be used as US was adjusted for each participant as described above. Finally, participants were informed that they had no effect on shock administration.

The experiment consisted in a modified version of a classical differential threat conditioning and extinction procedure^39,41,47^ (see Fig. 1). During the experiment, each trial consisted in the presentation of a context scene for 1 s, followed by one of the two CSs presented within the context scene for 4 s, and ending with the context scene still visible for 1 more second. The intertrial interval (ITI) was a white fixation cross on a black background, with a variable duration ranging from 11 to 16 s. The length of the ISI was chosen to avoid complete masking of conditioned SCRs by preceding unconditioned SCRs to the shock.

The experimental protocol was administered over two separate days. On Day 1, three different phases were presented: habituation, threat acquisition and threat extinction.

At the beginning of the session, participants were informed that different images would be presented on the screen, and the task of the participant would be to carefully observe the images, as some of them might be paired with the electrical stimulation.

The habituation phase included 4 trials, in which the CS+ and CS− (2 for each) were presented in random order either within the ‘danger’ context or the ‘safe’ context, to ensure the absence of any baseline differences within and between age groups in response to the CSs. Few habituation trials were used to avoid retardation of learning due to nonreinforced exposure to CS+ (the latent inhibition effect^48^). The threat acquisition phase consisted of 20 CS+ and 20 CS− trials, all presented within the ‘danger’ context (yellow-blue room or grey-red room). One CS (plant or lamp) was associated with the administration of a shock pulse, resulting in the conditioned stimulus (CS+), while the other CS was never paired with any consequence, resulting in the neutral stimulus (CS−). In CS + trials, the US (shock) was administered 60% of times (12 out of 20 trials), 3.8 s after the CS+ onset, and coterminated with the CS+. In CS− trials, the US was never administered. The trials were pseudo-randomly presented to participants such that no more of three identical CSs occurred in a row. During the extinction phase, which followed immediately, the CSs were presented within a distinct (‘safe’) context. In this phase, participants learned that the CS+ was no longer followed by the US. Both CS+ and CS− stimuli were presented 20 times without the US. Characteristics of CSs, trial order, and ITI were as in the acquisition phase.

On Day 2, (24 hr after the extinction phase), two additional phases were presented: extinction recall, and threat renewal, during which the ability to selectively retrieve extinction memory as a function of context (safe vs. dangerous) was tested. Participants were told that the procedure for this second part of the experiment would be the same as on the previous day. During extinction recall, 10 CS+ (without the US) and 10 CS− were presented within the ‘safe’ context, where extinction learning previously occurred. During threat renewal, 10 CS+ (without the US) and 10 CS− were presented within the danger context, where the original threat conditioning was learned. Stimulus and ITI timings were identical on Days 1 and 2.

To assess the acquisition of a conditioned response to CSs, SCR was measured during all the experimental phases, and the responses related to CS+ were contrasted against those related to CS−.

It has to be noted that shocks were delivered only in the acquisition phase of the first day and never delivered in all other phases of the experiment.

### SCR data analysis

SCR data were offline analyzed using custom-made MATLAB scripts, and all statistical analyses were performed with STATISTICA (Dell Software, released September 2015, StatSoft STATISTICA for Windows, version 13.0, Round Rock, Texas, USA). Assumption of normal distribution of data was verified. Mixed-design analyses of variance (ANOVAs) were used to investigate differences within and between age groups. Post-hoc analyses were conducted with Newman-Keuls test and the significance threshold was p < 0.05. Data were extracted from the continuous signal and calculated for each trial as the peak-to-peak amplitude of the largest deflection during the 0.5 to 4.5 s time window after stimulus onset. The minimum response criterion was 0.02, and smaller responses were encoded as zero. SCR following the US was analyzed to assess unconditioned responding, whereas SCR following the CS was analyzed to assess conditioned learning. Regarding SCR to the US, stimulus onset was represented by the time of shock administration; regarding SCR to CS, stimulus onset referred to the time of CS appearance. Raw SCR scores were square-root transformed to normalize the data distribution and scaled to each participant’s mean square-root-transformed US response, to account for inter-individual variability^49^. To reduce interindividual variability, raw scores were range corrected by dividing each individual score by the subject’s mean SCR response to US^50^. This procedure can reduce error variance, thus increasing statistical power when comparing groups of participants. In this way, conditioned responses can be directly compared across groups without confounding baseline differences in skin conductance levels^33^. Because after range correction the resulting distribution was positively skewed, these data were then square-root transformed prior to statistical analyses^51^.

Regarding the response to the US, mean SCRs to the 12 shocks were analyzed. Concerning the response to the CS, SCR data were collapsed into “early” and “late” trial blocks of each phase (threat acquisition and threat extinction on Day 1; extinction recall and threat renewal on Day 2), as learning typically varies across time within each learning phase.

On Day 1, to assess conditioned responses to the CS separated from unconditioned responses to the shocks themselves, only non-reinforced CS trials were analyzed. Learning-related changes were hypothesized to be found in the ‘late acquisition’ and ‘late extinction’ phases, as reported previously^39,41^.

### Subjective fear ratings and contingency awareness

Given the importance of controlling for the influence of explicit knowledge on conditioned learning to correctly interpret aging effects^33^, subjective measures of threat conditioning and extinction were also acquired. At the end of each phase (habituation, threat acquisition, extinction, extinction recall, threat renewal), participants were asked to report the level of fear experienced at the presentation of CS+ and CS− during the task, rating via an 11-point Likert scale (range 0–10) with anchor points “not at all fearful” and “extremely fearful”. The order of questions was counterbalanced across participants.

To ensure explicit awareness of threat conditioning and extinction, at the conclusion of acquisition and extinction phases (Day 1) participants were asked to explicitly indicate which CS (plant and lamp), and context scene (yellow-blue room and grey-red room), was more frequently associated with the electrical stimulation, by using a forced-choice recognition procedure (‘Was picture of lamp or picture of plant more often followed by the electrical stimulation?’).

## Results

### US intensity and unconditioned responding

One-way ANOVAs were used to evaluate differences in US intensity and mean SCR to the US. Results showed no difference in the intensity of shock pulses (F(1,46) = 0.08, p = 0.928) between young (mean = 7.49 mA, SD = 2.21 mA) and older (mean = 7.56 mA, SD = 2.62 mA) adults. Likewise, no difference between young (mean = 1.02 μS, SD = 0.16) and old (mean = 0.97 μS, SD = 0.14) group was found in the mean SCR in responses to US (F(1,46) = 0.781, p = 0.381). On average, therefore, the intensity of the electrical stimulation received by participants, the subjective quality of perception (“highly annoying, but not painful), as well as the physiological response to it (i.e., arousability) did not differ significantly between age groups.

### Habituation (Day 1)

To analyze habituation, a 2 × 2 repeated measure ANOVA was performed on SCR, with Group (young/old) as a between-subject factor, and Stimulus (CS+/CS−), as a within-subject factor.

Analysis showed no significant main effect of Group (F(1,46) = 0.23, p = 0.632, partial η^2^ = 0.02), Stimulus (F(1, 46) = 0.304, p = 0.58, partial η^2^ = 0.01), or Group by Stimulus interaction (F(1, 46) = 1.22, p = 0.277, partial η^2^ = 0.01), thus revealing that at baseline there were neither within group nor between group differences in orienting responses to the CS+ and CS−.

### Threat acquisition and extinction (Day 1)

**Figure 2:**
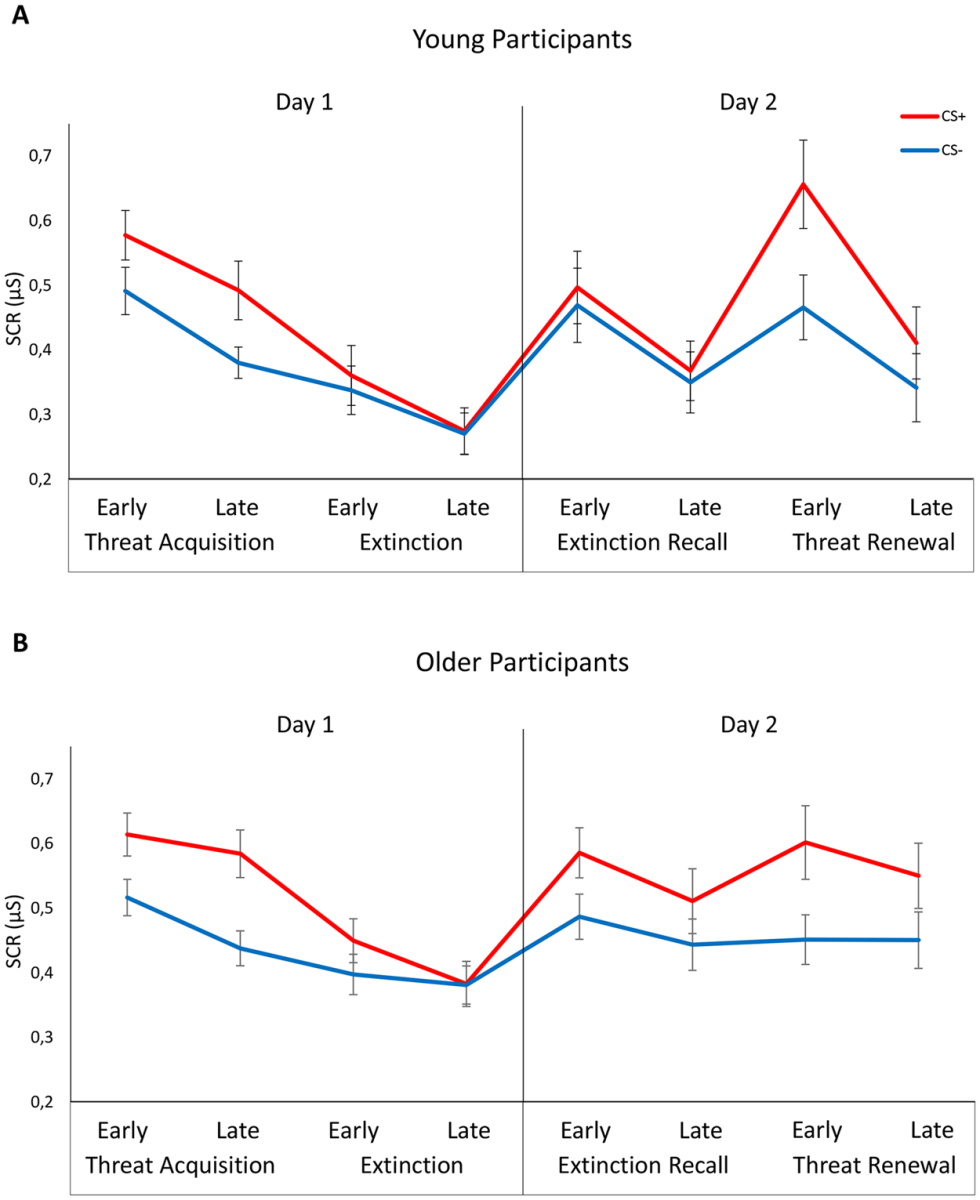
Skin conductance responses. Graphs illustrate mean skin conductance responses (SCRs) to the conditioned (CS+) and neutral (CS−) stimuli during early and late blocks, in young (**A**) and older (**B**) participants on Day 1 (threat acquisition and extinction phase) and Day 2 (extinction recall and threat renewal phase). Data demonstrate no effect of aging on threat acquisition and extinction on Day 1. In contrast, only older participants failed to recall the previous extinction in the safe context on Day 2, while young participants specifically adapted their conditioned responses according to the context. Error bars represent standard error.

To analyze SCR data recorded in Day 1, a 2 × 2 × 2 repeated measure ANOVA with Group (young/old) as a between-subject factor, and Stimulus (CS+/CS−), and Block (early/late) as within-subject factors was carried out separately for each phase (threat acquisition and extinction; see Fig. 2).

During threat acquisition, results showed a main effect of Stimulus (F(1,46) = 65.901, p < 0.001, η^2^ = 0.58), reflecting stronger responding to the CS+ (young group mean = 0.52 μS, SD = 0.17 μS; old group mean = 0.59 μS, SD = 0.14 μS) than to the CS− (young group mean = 0.43 μS, SD = 0.14 μS; old group mean = 0.47 μS, SD = 0.12 μS), and a main effect of Block (F(1,46) = 17.467, p < 0.001, η^2^ = 0.27), which reflected higher SCRs overall during late than early acquisition block. This result implies that that differential threat learning to the CS+ took place overall during the acquisition phase. Importantly, the analysis revealed neither a significant main effect of Group, nor interaction of Group with Stimulus or Block (all ps > 0.23), thereby suggesting that conditioned learning took place equivalently in young and older participants.

During extinction, analysis revealed a significant Stimulus by Block interaction (F(1,46) = 7.17, p = 0.010, η^2^ = 0.13), but no significant main effect or interactions with the factor Group (all ps > 0.08). Post-hoc analyses showed that participants had significantly stronger responses to CS+ than to CS− during early extinction (CS+: young group mean = 0.36 μS, SD = 0.22 μS; old group mean = 0.44 μS, SD = 0.16 μS; CS−: young group mean = 0.33 μS, SD = 0.18 μS; old group mean = 0.39 μS, SD = 0.15 μS), but SCR differences between CS+ (young group mean = 0.27 μS, SD = 0.17 μS; old group mean = 0.38 μS, SD = 0.17 μS) and CS− (young group mean = 0.27 μS, SD = 0.15 μS; old group mean = 0.38 μS, SD = 0.14 μS) disappeared for both groups during late extinction.

Thus, overall results showed equivalent responding of the two experimental groups across all three phases (i.e., habituation, threat acquisition, and extinction) of Day 1, prior to the extinction recall and threat renewal manipulations of Day 2.

### Extinction recall and threat renewal (Day 2)

To analyze SCR data collected in Day 2, a 2 × 2 × 2 repeated measure ANOVA with Group (young/old) as a between-subject factor, and Stimulus (CS+/CS−), and Block (early/late) as within-subject factors, was carried out separately for each phase (extinction recall and threat renewal; see Fig. 2).

During extinction recall, analysis showed a main effect of Stimulus (F(1,46) = 13.512, p = 0.001, η^2^ = 0.22), which reflects elevated responses to the CS+ (young group mean = 0.44 μS, SD = 0.24 μS; old group mean = 0.54 μS, SD = 0.21 μS) relative to CS− (young group mean = 0.41 μS, SD = 0.22 μS; old group mean = 0.46 μS, SD = 0.16 μS), and a main effect of Block (F(1,46) = 14.368, p = 0.001, η^2^ = 0.23), due to a progressive decrease of conditioned SCRs during the extinction recall phase in both groups. Crucially, the analysis revealed a significant Group by Stimulus interaction (F(1,46) = 4.401, p = 0.04, η^2^ = 0.087). Follow-up Newman-Keuls tests showed different pattern of SCRs between groups. Specifically, no difference in SCR was found during extinction recall in the young group (p = 0.27). In the old group, however, the SCR to CS+ was significantly higher than SCR to CS− (p < 0.001), demonstrating a return of threat response to previously extinguished CS+. Other comparisons were not statistically significant (ps > 0.31). Importantly, to control for the influence of depression and anxiety on extinction recall, we repeated the significant Group x Stimulus x Block analysis using ANCOVA with levels of depression and anxiety as additional covariates. The Group by Stimulus interaction remained statistically significant even after controlling for depression (F(1,45) = 3.981, p = 0.05, η^2^ = 0.082), and anxiety (F(1,45) = 4.798, p = 0.03, η^2^ = 0.096). Thus, aging was associated with impaired recall of extinction memory, both in the early and late portion of the phase.

During threat renewal, an ANOVA showed a significant main effect of Stimulus (F(1,46) = 38.551, p < 0.001, η^2^ = 0.456), indicating significantly greater SCRs associated to CS+ (young group mean = 0.53 μS, SD = 0.29 μS; old group mean = 0.57 μS, SD = 0.25 μS) than to CS− (young group mean = 0.40 μS, SD = 0.24 μS; old group mean = 0.45 μS, SD = 0.18 μS), in both groups. A main effect of Block (F(1,46) = 37.989, p < 0.001, η^2^ = 0.452), and a Group by Block interaction (F(1,46) = 21.535, p < 0.001, η^2^ = 0.318) were also found. This result reflected reduced SCRs overall during late than during early threat renewal in young (p < 0.001), but not in older (p = 0.287), adults. Importantly, neither a significant main effect of Group nor interaction of Group with Stimulus was found (all ps > 0.51). Therefore, both groups showed differential SCR to CS+ compared to CS− during renewal.

**Figure 3:**
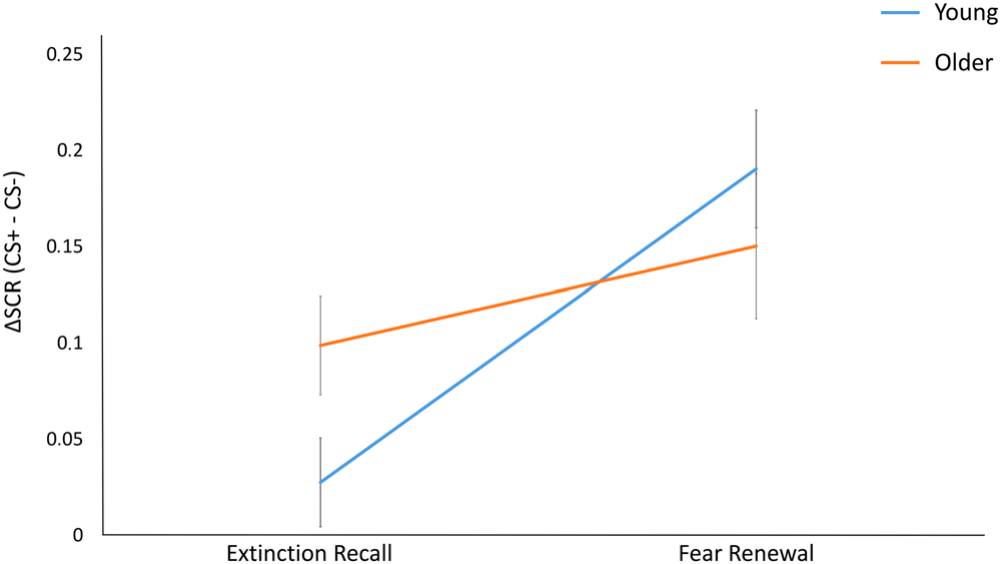
ΔSCR (calculated by subtracting SCR to CS− from SCR to CS+) during early extinction recall and threat renewal (Day 2). While young participants adjusted their psychophysiological response based on the context, old participants show a similar activation regardless of the contextual information. Error bars represent standard error.

To directly assess context-dependent modulation of extinction memory in young and older participants, the differential threat response (ΔSCR) was calculated by subtracting SCR to CS− from the SCR to CS+, both during early extinction recall and early threat renewal. Extinction recall analysis focused on the first block of trials (‘early extinction recall’) in order to avoid confounding extinction memory with new extinction learning taking place during the extinction recall phase itself^52^. For the same reason and to be consistent, threat renewal also focused on the first block of trials (‘early threat renewal’). An ANOVA, with Group (young/old) as a between-subject factor, and Phase (extinction recall/threat renewal) as within-subject factors, showed a main effect of Phase (F(1,46) = 22.108, p = 0.001, η^2^ = 0.47) and, more critically, a Phase by Group interaction (F(1,46) = 5.975, p = 0.018, η^2^ = 0.11). Follow-up Newman-Keuls tests revealed that the young adults showed normal context-sensitivity during extinction recall, with significantly lower ΔSCR in the extinction (safe) compared with the acquisition (danger) context (p = 0.012). In contrast, older adults did not demonstrate a significant effect of context on ΔSCR on Day 2 (p = 0.12). The Phase by Group interaction remained significant (p < 0.05) even after adjusting for the influence of depression and anxiety levels as additional covariates, suggesting that impaired context-dependent modulation of threat and extinction memories were mediated by aging, and not by depression or anxiety. These results (Fig. 3) suggest that on Day 2 young participants adapted their responses to threat based on the context in which the stimuli were presented. Differently, older participants did not recall extinction memory, responding specifically to CS+ regardless the context in which it was presented.

### Subjective fear ratings and contingency awareness

**Figure 4:**
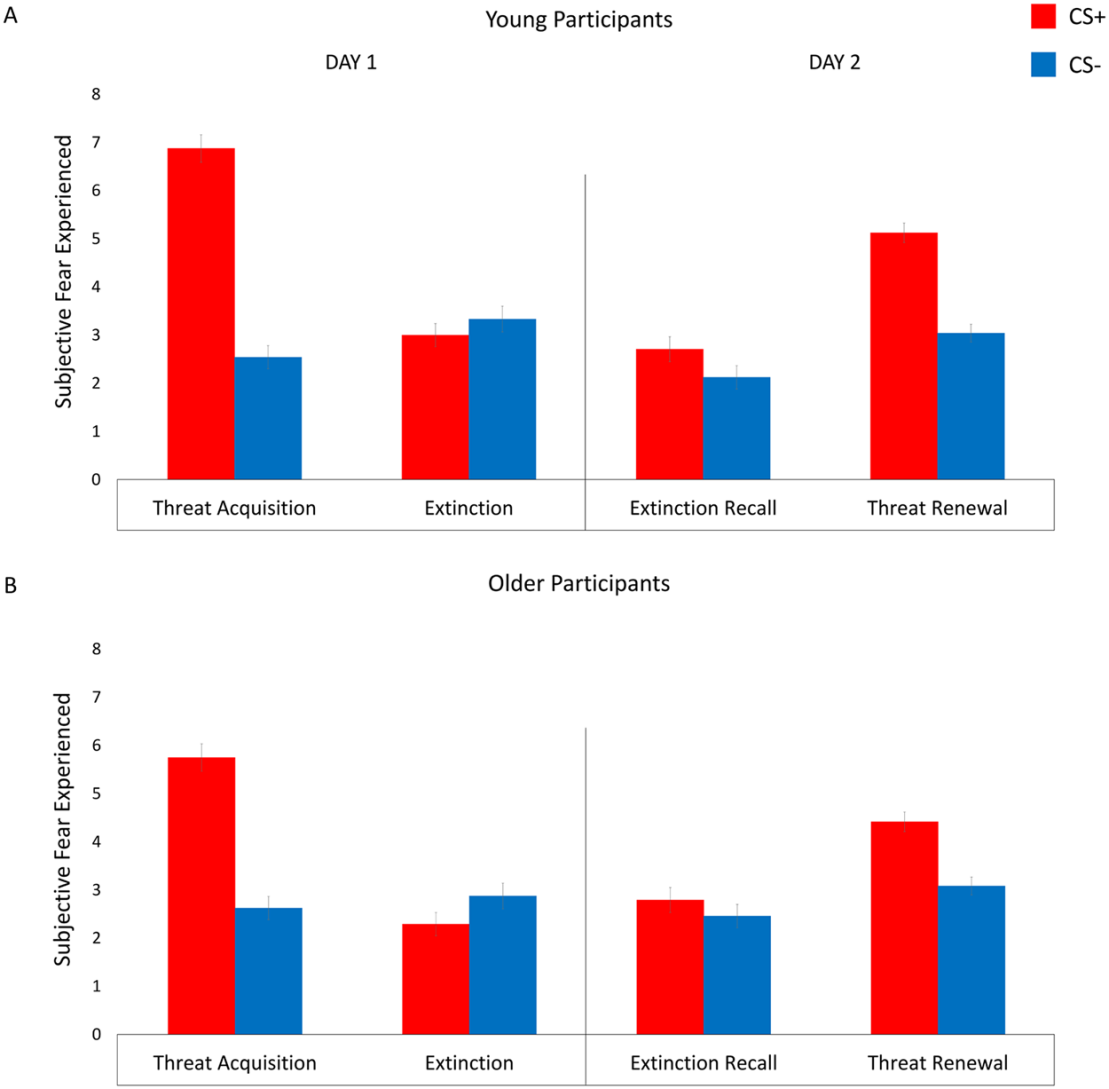
Subjective fear ratings. Graphs illustrate the level of self-reported fear to the conditioned stimuli during the experimental phases in young (**A**) and older (**B**) participants. Error bars represent standard error.

A 2 × 2 × 5 repeated measure ANOVA with Group (young/old) as between-subject factor, Stimulus (CS+/CS−) and Phase (habituation/acquisition/extinction/extinction recall/threat renewal) as within-subject factors, was used to assess participants’ fear ratings of conditioned stimuli in each experimental phase (Fig. 4). A significant Stimulus by Phase interaction (F(1,184) = 40.439, p < 0.001, η^2^ = 0.49) was found, indicating that self-report level of fear to CS+ and CS− differed depending on experimental phases. The Stimulus by Phase by Group interaction was not significant (F(1,184) = 1.081, p = 0.367, η^2^ = 0.02), indicating that the old group did not differ from the young group in the level of self-report fear to the conditioned stimuli during the experimental phases. Newman-Keuls test for the significant interaction showed that, in the habituation phase, self-report fear to CS+ (young group mean = 2.58, SD = 0.92; old group mean = 2.66, SD = 1.27) and CS− (young group mean = 2.87, SD = 1.22; old group mean = 2.79, SD = 1.41) were not significantly different (p = 0.846). Instead, in the acquisition phase, self-report fear to the CS+ (young group mean = 6.87, SD = 1.39; old group mean = 5.75, SD = 1.42) was significant higher than fear to the CS− (young group mean = 2.54, SD = 1.17; old group mean = 2.62, SD = 1.46; p < 0.001). During extinction, self-report fear to the CS+ (young group mean = 3, SD = 1.17; old group mean = 2.29, SD = 1.04) and CS− (young group mean = 3.33, SD = 1.30; old group mean = 2.87, SD = 1.19) were not significantly different (p = 0.967), as well as in the extinction recall phase (CS+, young group mean = 2.70, SD = 1.26; old group mean = 2.79, SD = 0.93; CS−, young group mean = 2.12, SD = 1.19; old group mean = 2.45, SD = 0.88; p = 0.506). Finally, during threat renewal, self-report fear to the CS+ (young group mean = 5.125, SD = 0.99; old group mean = 4.41, SD = 1.61) was significant higher compared to the CS− (young group mean = 3.04, SD = 0.90; old group mean = 3.08, SD = 1.24; p < 0.001).

Irrespective of the group, all participants correctly associated the context scenes with the administration of the electrical stimulation; moreover, 91% of young participants and 88% of older participants correctly paired the CSs with the corresponding outcome (p = 0.574). Thus, both young and older participants were able to verbally express CS-US, as well as context-US, contingencies.

### Neuropsychological assessment

Equivalent scores on each neuropsychological test were compared between young and older participants in the study and no significant differences were found (Table 1).

To further test the impact of neuropsychological variables, four separate stepwise regression analysis (forward selection) were performed on each task phase (threat acquisition/extinction/extinction recall/threat renewal). The raw scores of all neuropsychological tests were used as regressors (namely, Raven Progressive Matrices, Verbal Span, Prose Recall, Attentional Matrices Test, and Weigl’s Sorting Test) and the differential conditioned response (ΔSCR) was used as a dependent variable.

**Figure 5:**
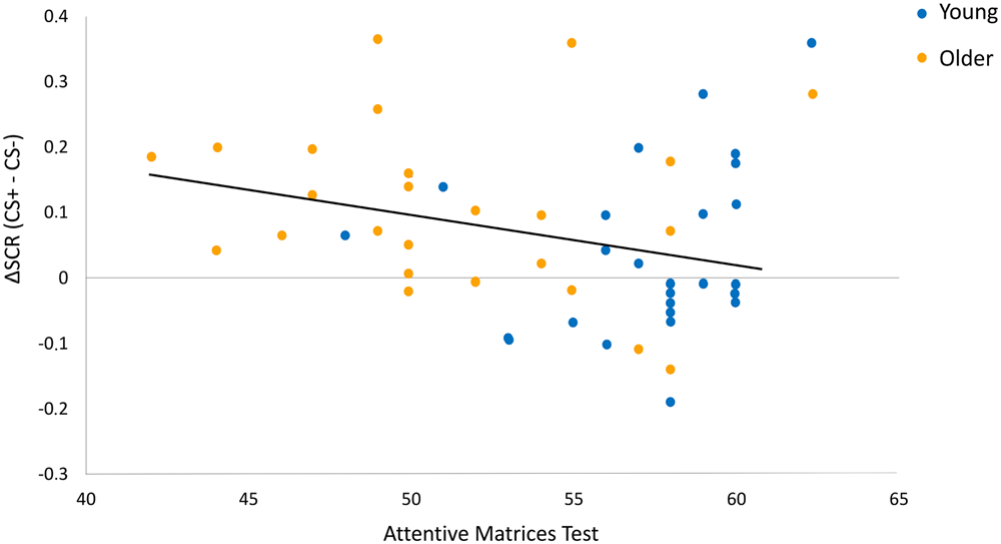
Impact of neuropsychological variables. Regression analysis reported a significant influence of selective visual attention, as assessed by the Attentional Matrices Test, on ΔSCR measured during early extinction recall phase.

For the extinction phase (Day 1), the best model (F(1,46) = 15.93, p < 0.001, R^2^ = 0.14) reported a significant effect only of Weigl’s Sorting Test (β = −0.37, t = −2.68, p = 0.01). For the extinction recall phase (Day 2), the best model (F(1,46) = 5.19, p = 0.03, R^2^ = 0.11) reported a significant effect only of the Attentional Matrices Test (β = −0.32, t = −2.27, p = 0.03) (Fig. 5). No significant effects were found for acquisition and threat renewal phases.

### Influences between acquisition and recall of threat and extinction

To test for a possible relation between the conditioned responses (ΔSCR) at threat learning and retrieval, a correlation between threat acquisition (Day 1) and threat renewal (Day 2), and a correlation between extinction (Day 1) and extinction recall (Day 2), were calculated separately within each group. Furthermore, to test for a possible relation between the conditioned response (ΔSCR) within each testing session, a correlation between threat acquisition and extinction (Day 1) and a correlation between threat renewal and extinction recall (Day 2), were calculated separately within each group. Pearson’s correlation coefficient and one-tailed, Bonferroni-corrected p-value are reported.

Young participants showed a trend in the correlation between threat acquisition and threat renewal, but this resulted non-significant when Bonferroni-corrected (r = 0.40, p > 1). No significant correlations were found between extinction and extinction recall (r = −0.02, p > 1), between threat acquisition and extinction (r = 0.09, p > 1), and between extinction recall and threat renewal (r = 0.006, p > 1) in this group.

Older participants showed a significant positive correlation between threat acquisition and threat renewal (r = 0.48, p = 0.024), and a significant positive correlation between extinction recall and threat renewal (r = 0.67, p < 0.01). No significant correlations were found between extinction and extinction recall (r = 0.25, p = 0.42), and between threat acquisition and extinction (r = 0.2, p = 0.32) in this group.

Taken together, these results seem to indicate that similar processes may be involved in the acquisition and renewal of a threat in older and, possibly, in young participants. This second interpretation, however, has to be taken cautiously, as this trend is visible, but not significant when applying a Bonferroni correction. However, extinction recall and threat renewal clearly seem to involve similar processes in old, but not in young participants.

## Discussion

Learning to disregard a stimulus that no longer predicts an aversive outcome, i.e., extinction, is critical for adaptive behaviour in a changing environment. Contextual information is particularly important in regulating the expression of responses to threat after these responses have been extinguished^3^. Declines in the ability to process contextual information may represent a fundamental mechanism of age-related cognitive changes^24^. The present study was the first to examine the influence of normal aging on context-dependent recall of extinction of responses to threat. Healthy young and old adults were tested in a multi-phase study over two days^39,52^. During the first day, participants were threat conditioned to two visual stimuli (CS+ and CS−) within a specific (danger) visual context, and then underwent threat extinction within a different (safe) context. On the second day, the ability to selectively recall extinction memory within these two different contexts (danger and fear) was assessed.

Results showed that young participants were able to use contextual information to flexibly guide their learned responses to threat (as expressed by SCR), whereas older participants showed impaired modulation of the responses by contextual information. More specifically, on the first day, all participants were equally able to acquire and completely extinguish a threat conditioned response (i.e., higher SCR to CS+ as compared to CS− during threat acquisition, and equal SCR to CS+ and CS− during extinction). On the second day, young participants showed a context-dependent modulation of the autonomic responses, as higher SCR to CS+, compared to CS−, was observed in the danger context, but not in the safe context^53^. In stark contrast, older adults showed an impaired context-guided recall of extinction, with higher SCR to CS+, as compared to CS− in both danger and safe context (Fig. 2).

These results are consistent with the presence of either a specific extinction recall deficit, or a more general context-processing deficit. Our finding that differential responding to the CS+ versus the CS− increased from the safe (extinction) to the danger (renewal) context in the young but not in the older participants strongly suggests that aging is associated with a more general loss of context sensitivity in memory expression (Fig. 3). Moreover, on Day 2, there was a significant positive correlation between differential threat responses in the safe (extinction recall) and danger (threat renewal) context in the older, but not in the young, adult group. This further suggests that aging is associated with loss of contextual control of extinction, causing extinguished threat memories to inappropriately renew in any context. Interestingly, all participants were equally able to learn and explicitly report the association between conditioned stimuli, context scenes, and aversive US (i.e. contingency awareness), as well as rate how fearful each stimulus was in each context (i.e., affect ratings), thus revealing that aging specifically precludes recall of extinction memories as indexed by physiological responses (Fig. 4).

The present findings were not related to differences in global autonomic responsivity, as unconditioned responses to the shock were the same across both groups. Likewise, results were unlikely due to changes in trait anxiety, or depressive conditions, since we did not find differences in these control variables between young and old participants. Regarding neuropsychological performance, it is important to note that all participants performed within the normal range compared with age and education-adjusted norms, and that the groups did not differ on age and education adjusted scores (Table 1). Therefore, the impairment in context-dependent extinction recall in older participants was not related to age-related cognitive decline (American Psychiatric Association, 1994).

Taken together, these findings indicate that older adults were less able to use contextual information to recall extinction memory and modulate the expression of the defensive responses to threat in a context-dependent manner, despite their preserved ability to acquire and extinguish a threat conditioned response.

Evidence of age-related changes in threat conditioning from non-human studies tend to report normal acquisition of simple forms of threat learning, but deficits in more complex aspects, such as acquisition and retention of contextual conditioning^31,54–57^. In line with the present findings, during tone threat conditioning, old mice exhibit a deficit in the use of context to modulate responses to threatening cues^32^. In particular, compared to young mice, aged mice showed low levels of threat responses regardless of the context, whereas young mice demonstrated context-dependent expression of renewal of responses^32^. Remarkably, both threat conditioning and immediate extinction were similar in the two groups.

In humans, LaBar and colleagues^33^ suggested an age-related impairment in threat conditioning as secondary to poor CS-US contingency awareness. More specifically, they found an age-related impairment in the expression of both threat conditioned responses and discriminative conditioning accounted for by a lack of awareness of the CS-US contingencies. Although awareness is neither necessary nor sufficient for normal conditioning learning^58,59^, it may play an important role in complex learning paradigms. Age effects may be at least partially due to a higher number of unaware subjects in old populations^60^, and it seems likely that old participants have more problems in recognizing the rule predicting US presentation during acquisition^59^. The present results show that older participants had threat acquisition and explicit awareness of CS-US and context-US contingencies comparable to those of young participants. As such, results of the present study are in line with LaBar^33^ findings in showing no age-related reductions of threat learning and extinction when contingency awareness is controlled. Thus, the failure in context-dependent extinction recall we observed in older participants does not seem to be due to a general learning deficit, or to a lack of contingency awareness and explicit knowledge acquired during the task.

In Pavlovian conditioning, the context is often referred to as an “occasion setter”, that is a modulating stimulus whose role is to disambiguate the current meaning of the conditioned stimulus^4,61^. Thus, in extinction procedures, context serves as an occasion setter that favours retrieval of the ‘safe’ CS–no US memory in the extinction context, and the ‘fearful’ CS–US memory in the acquisition (or any other) context^62,63^, which in turn inhibits and excites, respectively, the conditioned response^64^. In older adults, the persistence of conditioned responses in the extinction context indicates an inability to correctly use contextual information to modulate responses to threat. In other words, in older participants, the context appears not able to operate as a gate that disambiguates the CS’s current relation with the US stimulus^61,65^. Current theorizing in cognitive aging offers a wide variety of accounts for performance decline in context processing and its utilization as occasion setter, including poor distribution of attentional resources^24,66^, reduction in working-memory capacity^67,68^, and failure of inhibitory processes^69^. These represent distinct but highly interdependent mechanisms that may influence each other^70^. Importantly, the present study found that the magnitude of the psychophysiological index of extinction recall was positively correlated with accuracy in the attentive matrices test (Fig. 5), a visual search task thought to index selective visual attention^45,71^. That is, individual and age-related differences in selective attention performance predicted subsequent context-dependent recall of extinction memory. Thus, we tentatively suggest that age-related declines in the efficiency of selective attention, possible due to a age-related reduction in available processing resources^72^, may lead to weak representation of contextual information and reduced ability to encode the appropriate CS-context relationship, thus promoting overgeneralization of threat responses to many contexts in older adults. These results are consistent with emerging theories that age-related declines in processing contextual information are attributable to poorer selective attention and/or greater inhibitory deficits in older adults^73^. Additional research is certainly still warranted, however, that directly examines the relationship between selective attention and context dependency of extinction in young and older adults.

Although the present study did not directly investigate the neural substrates of threat conditioning and extinction in aging, deficit of context-guided recall of extinction may be linked to age-related changes in the neural structures underpinning context-dependent behaviour^74^. Studies in animals support the view that a neural circuit that involves the hippocampus and medial prefrontal cortex is essential for contextual retrieval of threat and extinction memories^20,75^. Consistent with this view, brain imaging studies in humans^10,39^ reported that the ventromedial prefrontal–hippocampal network is selectively involved in context-dependent regulation of extinction and threat memories. More specifically, during recall of extinction memory, the medial prefrontal cortex would act to inhibit the amygdala, preventing a response to threat, based on contextual information provided by the hippocampus^9,10,76^. There is substantial evidence that a number of structural and physiological alterations preferentially influence the prefrontal cortex and medial temporal lobe in advanced aging, even in the absence of disease^22,77,78^. These disruptive brain changes may underlie impairments in context-dependent extinction recall, as well as cause the decreased efficiency with which older adults use contextual information to determine when and where it is appropriate to express fear. Additional research will be needed to clarify the underlying neuroanatomical mechanisms of extinction recall and context processing deficits in aging, providing important clues to the pathophysiology of these disorders. Moreover, such data could help to advance our understanding of the neural mechanisms underlying behavioural therapy, such as exposure therapy^79,80^, aimed at limiting pathological fear.

The results of this study are tempered by a number of limitations. First, the present study used mildly aversive electro-tactile stimulation as US. Since differences in threat learning and extinction may derive from differences in US reactivity, there is the need to replicate these results with a different type of US, for instance, aversive auditory stimuli, such loud noise or complex human scream. Second, extinction recall and threat renewal were both tested at a single time-point after extinction learning (24 hr later, on Day 2). Future studies should also vary the interval between extinction training and recall/renewal testing, to determine whether aging may interfere with, or simply delay, the consolidation process of extinction memories. Third, we obtained one set of subjective measures following each phase of the study rather than continuous assessment. Online (i.e., trial-by-trial) measures could be used in future studies to provide a more accurate assessment of US expectancy and CS valence during learning and extinction. Note, however, that in older individuals the value of including ratings during the experimental learning phases should be carefully balanced against the possible impact of rating procedures on attention and executive resources, which in turn may affect the time course and strength of threat conditioning^81^.

In conclusion, the present study documented the influence of normal aging on context-dependent recall of conditoned emotional respones. Contextual processing is especially vulnerable to advanced aging^70,82,83^. In line with this, the present data showed that (a) young and older participants were equally able to acquire and extinguish an autonomic conditioned response to threat, and that (b) older participants failed to modulate such response based on a context-driven retrieval of threat memories, raising the possibility that their extinction recall deficit is a consequence of a more general impairment in using contextual information. This lack of flexible adaptation to contextual cues may play a role in the development of late-onset anxiety disorders^84^, due to neural alterations that normally accompany healthy aging, particularly in the frontal and medial temporal lobes^85^. However, there is still a need for studies directly linking together the use of contextual information for flexible responses to threat, and age-related alterations of relevant neural structures underpinning aversive learning and memory processes.
